# Time to include pandemic preparedness training to healthcare curriculum

**DOI:** 10.1080/10872981.2020.1820229

**Published:** 2020-09-14

**Authors:** Usama Farghaly Omar, Jacquilyne Kharlukhi, Arun-Kumar Kaliya-Perumal

**Affiliations:** a Department of Orthopaedic Surgery, Khoo Teck Puat Hospital, Singapore; b Department of Paediatrics, Sree Balaji Medical College and Hospital, Chennai, TN, India; c Nanyang Technological University Singapore, Singapore

**Keywords:** COVID, coronavirus, healthcare curriculum, infectious disease, pandemic

COVID-19 is still on the rise. Even though tight measures are in place to control the spread, it’s being a tough equation to be wisely solved. In this pandemic situation, priorities include not just protecting the community, but also protecting frontline healthcare personnel who are at a higher risk of being infected. Morbidity among healthcare personnel is something that could completely collapse the health service; hence, all necessary steps should be taken to avoid this grave situation. While it is important that frontline healthcare personnel are provided with necessary personal protective equipment, it is equally important that they have adequate training to use them appropriately.

Reports from around the world suggest that a significant number of healthcare personnel have become infected and some of them have died [1,2]. Moreover, this is not the first time that a pandemic virus has infected the frontline healthcare personnel in large. If we look back in history, Severe Acute Respiratory Syndrome (SARS) was among the most recognizable outbreaks that badly affected the healthcare personnel [3]. In these kinds of situations, those healthcare personnel who have contracted the disease, or those who have been in close contact (without personal protective equipment [PPE]) with a positive case, need to be under quarantine for a specific period and test negative before they return to work [4].

This leads to a shortage of healthcare personnel and a global urge for their deployment to cope with an aggressive increase in case numbers. Today, hospitals have managed this shortage by diverting most of the available healthcare personnel to COVID-19 tasks and also by stepping up students and those in junior posts [5]. This has drawn attention on the need for being skilled to work in the frontlines during a pandemic situation. While only personnel from certain specialities such as emergency medicine and communicable diseases, who represent the main frontline have this training as a part of their curriculum, others must undergo this training before being deployed to manage such tasks. Hence it is better to incorporate this training to the curriculum of all healthcare professionals.

This pandemic preparedness training should be in addition to the Basic Life Support (BLS) and Advanced Cardiac Life Support (ACLS) training that are now in practice. Under this programme, trainees should have practical sessions on how to deal with an infectious disease outbreak both in the community and at hospital levels. Trainees should understand how to properly use PPE and how to provide resuscitation for a patient with a communicable disease. Recent recommendations have suggested that general guidelines for resuscitation may not apply during a pandemic situation like COVID-19 and that it needs certain modifications [6]. In Italy, they have developed and experimented a model for providing ventilation during CPR trough a supraglottic device with no exposure to patient’s direct exhaled gases as the patient is covered with adhesive sheets [7]. Trainees can be made aware of these kinds of alternatives if such are validated.

In most countries, owing to the COVID-19 experience, their Center for Disease Control and Prevention (CDC), run by the government, has standardized that all healthcare personnel should be well trained for using PPE and be ready for deployment. However, since currently no meetings or workshops are recommended in compliance with the safe distancing measures, CDCs could only provide guidelines and remote training using online platforms [8]. Had there been prior training, it would have been easier. Hence, it appears that there is a need to relook into hospital policies regarding compulsory pandemic preparedness training and requirements for healthcare personnel at all levels including students. If all current healthcare personnel and incoming students are trained, reallocating of services during a pandemic situation can be done with ease. This is another lesson this COVID-19 pandemic has taught us. It is time to include pandemic preparedness training to our healthcare curriculum.
